# Immune Thrombocytopenia Purpura Secondary to COVID-19

**DOI:** 10.7759/cureus.9083

**Published:** 2020-07-09

**Authors:** Joseph Bennett, Christopher Brown, Michael Rouse, Marc Hoffmann, Zhan Ye

**Affiliations:** 1 Internal Medicine, University of Kansas Medical Center, Kansas City, USA; 2 Hematologic Malignancies and Cellular Therapeutics, University of Kansas Medical Center, Kansas City, USA; 3 Pathology and Laboratory Medicine, University of Kansas Medical Center, Kansas City, USA

**Keywords:** immune thrombocytopenia purpura, covid-19, thrombocytopenia

## Abstract

A 73-year-old female with past medical history of essential hypertension, hyperlipidemia, seasonal allergies, and chronic back pain presented to the hospital with complaints of headaches, fevers, fatigue, generalized body aches, shortness of breath, and diarrhea. Initial complete blood count was remarkable for leukopenia with an absolute lymph count of 0.60 K/µL and severe thrombocytopenia (platelet count < 3 K/µL). She was tested for COVID-19 via nasopharyngeal swab polymerase chain reaction (PCR) testing and found positive. Additional labs showed an elevated D-dimer, C-reactive protein, fibrinogen, and lactate dehydrogenase. Vitamin B12 and folate levels were obtained and found to be normal. Peripheral smear showed no schistocytes or additional hematologic abnormalities apart from thrombocytopenia. The patient was transfused one unit of platelets with no improvement in platelet count. Fibrinogen count was obtained and found in normal range at 458 mg/dL. Prothrombin time (PT), activated partial thromboplastin time (aPTT), and international normalized ratio (INR) were all found to be normal. Immune thrombocytopenia purpura (ITP) was suspected and intravenous immunoglobulin (IVIG) was administered at a dose of 1 g/kg/day for two doses. By day 4, the patient had marked response to treatment with platelet recovery to 105 K/µL and subsequently discharged by day 5 with complete resolution of symptoms and platelet count of 146 K/µL. Twenty-eight days after discharge, she presented to hematology clinic with platelet count of 8 K/µL. Repeat nasopharyngeal swab PCR COVID testing was negative and she was treated with IVIG and pulse dexamethasone with prompt response, confirming suspicion of underlying, undiagnosed ITP prior to COVID infection.

## Introduction

Severe acute respiratory syndrome coronavirus 2 (SARSCoV-2), COVID-19, is most widely characterized by the presence of fever, cough, and respiratory distress, but is increasingly recognized to carry systemic complications [1]. Many cases have been found to display significant hematologic and thrombotic complications. The hematologic derangements are not insignificant, with increased risk for coagulation dysregulation and disseminated intravascular coagulation (DIC) [2-4]. Exacerbations of immune thrombocytopenia purpura (ITP) are common in patients with viral syndromes. However, ITP exacerbation in the context of COVID-19 infection is a rare, but increasingly recognized phenomenon [5]. Here, we provide a unique case of ITP, suggestive due to COVID-19 infection. This case illustrates the tenants of thrombocytopenia evaluation in addition to management in rare cases such as this. 

## Case presentation

A 73-year-old female with past medical history of hypertension and hyperlipidemia presented to the hospital with concerns of fever, shortness of breath, and diarrhea. Symptoms began the day prior to admission. She reported symptoms of fatigue, body aches, rhinorrhea, and a fever of 101°F while at home. She also reported a cough. Of note, the patient had traveled outside of the state the week prior to admission. Her husband had recently been sick with pneumonia and she was caring for him while at home. However, his COVID status was unknown. Initial exam was remarkable for a pleasant female who appeared clinically ill with stable vital signs and without clinical stigmata of severe thrombocytopenia. Complete blood count was obtained and showed hemoglobin of 10.5 gm/dL and undetectable platelets at < 3 K/µL. White blood cell count was found depressed at 4.1 K/µL with a depressed absolute lymph count of 0.60 K/µL. Cell counts reviewed from the year prior were within normal limits. Lactate dehydrogenase (LDH) was found elevated at 299 U/L. ADAMTS13 activity was found > 100% (reference range > 70%). Ferritin level was also elevated at 441 ng/mL. Additional work-up included vitamin B12 of 718 pg/mL and serum folate 22.3 ng/mL (reference range > 3.9 ng/mL). The patient was tested for COVID-19 via nasopharyngeal swab, which returned positive. Initial chest radiography was negative for any acute cardiopulmonary process. Blood cultures were obtained but remained no growth throughout admission.

**Figure 1:**
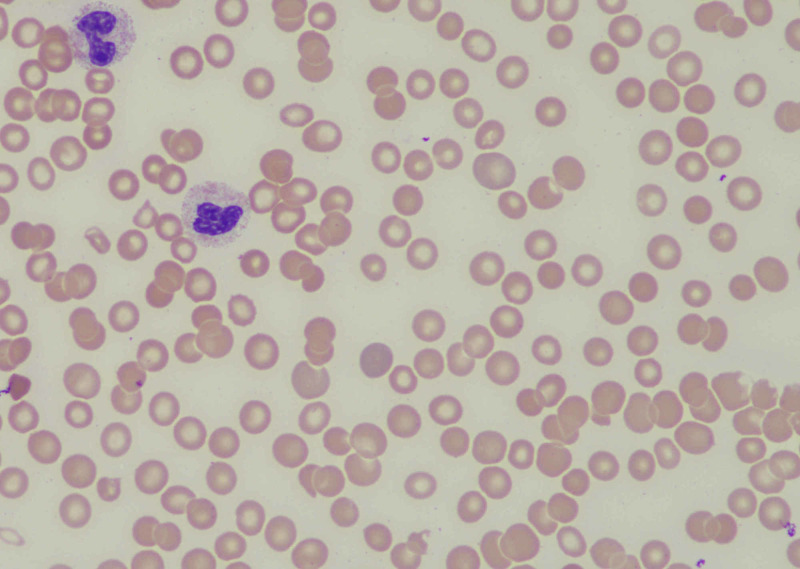
Peripheral smear demonstrating thrombocytopenia and normal platelet morphology

A peripheral smear was obtained and showed known thrombocytopenia, but no other hematologic features of diagnostic significance (Figure 1).

Additional labs included international normalized ratio (INR) 1.2, fibrinogen of 458 mg/dL (reference range 200-400 mg/dL), and immature platelet fraction of 4.9%. Activated partial thromboplastin time (aPTT) was obtained and found normal at 30 seconds. The patient was transfused one unit of apheresis platelets with no improvement seen in platelet count. ITP was suspected, and hematology was consulted. Intravenous immunoglobulin (IVIG) was administered at a dose of 1 g/kg/day for two doses. Corticosteroid administration was considered but not administered due to limited data availability and concerns of that may worsen outcomes during the active replicative phase of COVID-19.

Initial management, with exception of initiation of IVIG, was largely supportive in nature. However, due to worsening dry cough and ongoing gastrointestinal symptoms (significant diarrhea and abdominal discomfort), the patient was initiated on hydroxychloroquine (400 mg orally twice daily [day 1] followed by 200 mg twice daily [day 2-5]) and losartan 25 mg daily. A QTc of 375 ms was noted on the electrocardiogram (ECG) prior to initiating hydroxychloroquine. The patient responded well to the IVIG administration with an increase in platelet count to 105 K/µL on day 3 of admission. Despite initial management as described above, diarrhea and abdominal discomfort continued. Work-up included negative Clostridium difficile, giardia, Cryptosporidium, and rotavirus testing. The patient was started on an anti-diarrhea regimen (Lomotil® with PRN Imodium®). Respiratory and gastrointestinal symptoms resolved by day 4 of hospital admission. On the day of discharge, platelet count had increased to 146 K/µL. Twenty-eight days after discharge, she presented to hematology clinic with a platelet count of 8 K/µL. Repeat nasopharyngeal swab polymerase chain reaction (PCR) COVID testing was negative and she was treated with IVIG and pulse dexamethasone with prompt response, confirming suspicion of underlying, undiagnosed ITP prior to COVID infection.

## Discussion

The coronavirus pandemic of 2019, COVID-19, is rapidly evolving with unprecedented ramifications for health systems and individuals alike. SARS-CoV-2, presumed to have originated in Wuhan, has subsequently spread throughout China and is now a global pandemic [1]. With over 4,400,000 confirmed cases, the disease pathology and clinical manifestations have been documented with an abundance of literature [6]. A wide range in clinical presentation exist, ranging from mild symptoms to sepsis and death. A severe form of pneumonia is not an uncommon occurrence and is estimated to occur in up to 10%-15% of patients [7]. The hematologic manifestations of COVID-19 have emerged as a particularly important issue, as patients have been found to have clinical and laboratory findings which include thrombocytopenia, elevated D-dimer, prolonged prothrombin time, and DIC [2]. ITP in the setting of COVID-19 is an increasingly recognized entity. The first case was described in New England Journal of Medicine [5].

There is emerging data that COVID-19 carries particular risk for thrombotic complications in infected patients. Thrombocytopenia and elevated D-dimer have been seen in 36.2% of patients and 46.4% of patients, respectively, with higher rates occurring in severe disease [8]. Additional hematologic abnormalities include lymphocytopenia in 83.2% of patients and leukopenia in 33.7% of patients [8]. It is becoming evident that patients with COVID 19 are at increased risk of developing DIC [2-4]. Elevated D-dimer, elevated fibrin degradation products, and prolonged prothrombin time have all been shown carry confer a worse prognosis in infected patients [4].

Our case offers a unique difference to these findings, in ITP-related COVID-19 infection, and the absence of DIC. ITP is a diagnosis of exclusion in the evaluation of thrombocytopenia. While the pathogenesis is incompletely understood, the prevailing theory is that viral antigens cross-react with normal platelet antigens in a form of molecular mimicry causing platelet destruction. Some of the more common viruses that cause this phenomenon include varicella-zoster virus (VZV), HIV, hepatitis C, and cytomegalovirus [9-12]. It is possible that a similar mechanism exists with COVID-19, or COVID-19 may precipitate an exacerbation of preceding low-grade ITP disorder. One of the limitations of our report is the patient did not undergo testing for HIV or hepatitis, albeit clinical suspicion was low due the presence of an alternative viral etiology. In addition, a bone marrow evaluation was not obtained due to the temporal response to treatment and confirmation of diagnosis.

Empiric data are still limited regarding best practices regarding COVID-19. Our patient was initially on an angiotensin-converting enzyme (ACE) inhibitor prior to admission. She was switched to losartan due to theoretical benefits associated with angiotensin receptor blockers [13,14].

Previously, during the COVID-19 pandemic, corticosteroid administration was not recommended by either the CDC or WHO for the treatment of COVID-19 [15,16]. Historically, use of corticosteroid use in the setting of SARS-coV and the Middle East respiratory syndrome coronavirus (MERS-CoV) has been shown to worsen immune response and cause diffuse alveolar damage [17]. However, the RECOVERY (Randomised Evaluation of COVid-19 thERapY) trial showed that dexamethasone reduced deaths by one-third in ventilated patients (rate ratio 0.65 [95% confidence interval 0.48 to 0.88]; p=0.0003) and by one-fifth in other patients receiving oxygen only (0.80 [0.67 to 0.96]; p=0.0021) [18]. The above article is preprint and undergoing peer review at the time of this writing. Thus, clinicians should still take an individualized approach to patient care in the evaluation of risks and benefits when initiating corticosteroid treatments. 

In sum, it is important to consider the extrapulmonary manifestations of COVID-19 infection. While thrombotic complications are frequently considered, ITP is a rare and unique manifestation of the disease. The response to treatment methods and exclusion of secondary causes suggest that COVID-19 was the inciting factor of ITP in this case.

## Conclusions

Hematologic manifestations of COVID-19 are not infrequent and can carry life-threatening complications. Thrombocytopenia should always include a thorough evaluation for primary and secondary causes. ITP can often be unmasked or exacerbated by viral syndromes. While molecular mimicry is common with several viruses, it is possible that COVID-19 can induce a similar physiologic response. It is important for practitioners to be vigilant and aware of this phenomenon. 
